# Perioperative Blood Transfusion Promotes Worse Outcomes of Bladder Cancer after Radical Cystectomy: A Systematic Review and Meta-Analysis

**DOI:** 10.1371/journal.pone.0130122

**Published:** 2015-06-16

**Authors:** You-Lin Wang, Bo Jiang, Fu-Fen Yin, Hao-Qing Shi, Xiao-Dong Xu, Shuai-Shuai Zheng, Shuai Wu, Si-Chuan Hou

**Affiliations:** 1 Department of Urology, Qingdao Municipal Hospital, School of Medicine, Qingdao University, Qingdao, China; 2 Department of Urology, Qingdao Municipal Hospital, Dalian Medical University, Dalian, China; 3 Department of Obstetrics and Gynecology, Affiliate Hospital of Qingdao University, Qingdao, China; 4 Department of Urology, Affiliate Hospital of Qingdao University, Qingdao, China; Genentech Inc., UNITED STATES

## Abstract

**Background:**

Multiple studies have investigated the effect of perioperative blood transfusion (PBT) for patients with radical cystectomy (RC), but the results have been inconsistent. We conducted a systematic review and meta-analysis to investigate the relationship between PBT and the clinical outcomes of RC patients.

**Methods:**

We searched MEDLINE, EMBASE, the Cochrane library and BIOSIS previews to identify relevant literature for studies that focused on the relationship of PBT and outcomes of patients undergoing RC. A fixed or random effects model was used in this meta-analysis to calculate the pooled hazard ratio (HR) with 95% confidence intervals (CIs).

**Results:**

A total of 7080 patients in 6 studies matched the selection criteria. Aggregation of the data suggested that PBT in patients who underwent RC correlated with increased all-cause mortality, cancer-specific mortality and cancer recurrence. The combined HRs were 1.19 (n = 6 studies, 95% CI: 1.11–1.27, Z = 4.71, P<0.00001), 1.17 (n = 4 studies, 95% CI: 1.06–1.30, Z = 3.06, P = 0.002), 1.14 (n = 3 studies, 95% CI: 1.03–1.27, Z = 2.50, P = 0.01), respectively. The all-cause mortality associated with PBT did not vary by the characteristics of the study, including number of study participants, follow-up period and the median blood transfusion ratio of the study.

**Conclusion:**

Our data showed that PBT significantly increased the risks of all-cause mortality, cancer-specific mortality and cancer recurrence in patients undergoing RC for bladder cancer.

## Introduction

Bladder cancer is the fifth most common cancer in Western countries and the highest cause of death among urinary malignancies in China [1]. RC remains the gold standard for treatment of muscle-invasive bladder cancer. This procedure is associated with significant blood loss and a common transfusion requirement. Substantial improvements in surgical techniques and perioperative management in the past two decades have markedly reduced operative and hospital mortality rates for patients with bladder carcinoma undergoing cystectomy [2, 3]. However, a large number of patients still require perioperative BTs (PBTs).

Recently, many investigators have focused on the underlying association between PBT and outcomes in various operations, such as lung cancer [4, 5], hepatocellular carcinoma [6, 7], colorectal cancer [8, 9], and prostate cancer [10], but their results were highly contradictory. A previous meta-analysis performed by Liu et al [11] demonstrated that allogeneic blood transfusion (ABT) was associated with adverse clinical outcomes for hepatocellular carcinoma patients undergoing surgery, including increased death, recurrence and complications. PBT in patients with bladder cancer was associated with increased morbidity and mortality after RC in several observational studies [12, 13], but other studies [14, 15] did not show this association in multivariable analysis. Some studies indicated that the disease characteristics (e.g., older age, higher pathological stage, longer surgical time and greater estimated blood loss) of patients who received PBT, rather than PBT itself, lead to worse outcomes [14, 15]. However, no meta-analysis has focused on the association between PBT and outcomes after RC for bladder cancer. We performed a meta-analysis of eligible studies to investigate the relationship between PBT and the clinical outcomes of RC and clarify the exact impact of PBT in patients who have undergone RC.

## Methods

### 2.1 Data sources

We conducted a systematic literature search of MEDLINE, EMBASE, the Cochrane library and BIOSIS databases for studies that were published from the time of inception to October 2014 using terms such as ‘‘bladder and transfusion” and “cystectomy and transfusion” combined with Boolean operators where appropriate. We also searched the reference lists of relevant studies and previous meta-analyses for additional studies. Unpublished conference papers were screened from the ISI Web of Knowledge Conference Proceedings to ensure the search was as comprehensive as possible, and these data were also included when possible.

### 2.2 Study selection

Two investigators (Y-L.W. and F-F.Y) independently extracted data from eligible studies. Disagreements were resolved by discussion and consensus. Two investigators reviewed all studies that met the inclusion and exclusion criteria. The following information was recorded for each study: (1) the study had to report the correlation between perioperative allogeneic blood transfusion and outcomes in patients undergoing RC; and (2) data were available on clinical outcomes (e.g., all-cause mortality, cancer-specific mortality, or disease recurrence). We excluded reviews, letters without original data and editorials. For duplicate publications reported by the same authors, either the higher quality or most recent publication was selected.

### 2.3 Data extraction and quality assessment

Two investigators independently reviewed each eligible article and extracted information from all of the publications that met the inclusion criteria. Disagreements were resolved by discussion among all authors. The HR with 95% CI for all-cause mortality or cancer-specific mortality was extracted. The HR was extracted from the multivariable analysis when both univariable and multivariable analyses were available. The following relevant characteristics were listed: (1) first author’s name, (2) year of publication, (3) country of origin, (4) number of patients, (5) age of the patients, (6) characteristics of the study population, (7) disease stage, (8) period of follow-up, (9) period of recruitment, and (10) PBT rate (Table 1). All relevant texts, tables and figures were reviewed for data extraction.

**Table 1:** Characteristics of the included studies for the meta-analysis.

Author	Publication year	Country	Recruitment period	Follow-up, months	Study type	Number of patients	PBT rate	Age (mean, Y)	Stage pT1/pT2/≥pT3	NOS
**Sadeghi**	2012	USA	1989–2010	25.5	retrospective	638	32.80%	67.6	206/98//294	6
**Morgan**	2013	USA	2000–2008	25	retrospective	777	41.60%	69.5	250/196/331	6
**Linder**	2013	USA	1980–2005	130.8	retrospective	2060	62%	69	883/314/564	7
**Kluth**	2014	Multination	1998–2010	36.1	retrospective	2895	39%	67	879/681/1335	7
**Gierth**	2014	Germany	1995–2010	70.1	retrospective	350	63%	68	122/60/168	7
**Abel**	2014	USA	2003–2012	18.7	retrospective	360	67%	67.9	83/79/149	6

Abbreviations: PBT, Perioperative blood transfusion; NOS, Newcastle-Ottawa Scale

The study quality of retrospective studies was assessed independently by two reviewers with consideration of the following aspects of the Newcastle-Ottawa quality assessment scale cohort studies [16]. The scale focuses on three factors: Selection, Comparability and Exposure. We identified ‘high’ quality choices with a ‘star.’ A study could be awarded a maximum of one star for each numbered item within the Selection and Exposure categories. A maximum of two stars can be given for Comparability. Studies with a score equal to or higher than 6 were considered high quality.

### 2.4 Statistical analysis

A forest plot was utilized to aggregate HRs from the multivariate analyses of individual studies in a summary HR of the effect of PBT on mortality and recurrence. Percent weights given to each study was assessed by weight analysis. Heterogeneity among the outcomes of enrolled studies in this meta-analysis was evaluated by using Chi-square based Q statistical test. And I^2^ statistic was calculated to quantify the total variation consistent with inter-study heterogeneity, ranging from 0% to 100% Heterogeneity was significant and unacceptable while I^2^ statistic was greater than 50% [17]. P < 0.05 in Q statistical test was considered statistically significant. In the case of I^2^>50%, the summary HR and the accompanying 95% CI were calculated with a random effects model, whereas we used a fixed effects model in the case of low heterogeneity as defined by I^2^≤50%. We examined publication bias with the aid of a funnel plot [18], and a roughly symmetrical distribution on either side of the summary estimate suggested a lack of bias. Statistical significance was defined at the level of 0.05. All statistics were performed with the Review Manager software (version 5.3.3; Cochrane collaboration, http:ims.cochrane.org/revman/download).

We performed subgroup analyses to explore associations between characteristics (follow-up period, the number of study participants and BT ratio) of the studies and their results. Given that there are no strict standard cut-off point for each characteristic, we attempt to stratify our included studies for binary variable specifications achieved as close as possible balanced distributions. Thus, we determined the medians of follow-up period (>30.8 or ≤30.8 month), the number of study participants (>708 or ≤708), and BT ratio (>51.8% or ≤51.8%) as the cut-off points. We also performed sensitivity analyses to assess the influence of individual studies on the pooled HR of all-cause mortality.

## Results

### 3.1 Study selection and characteristics

**Fig 1:**
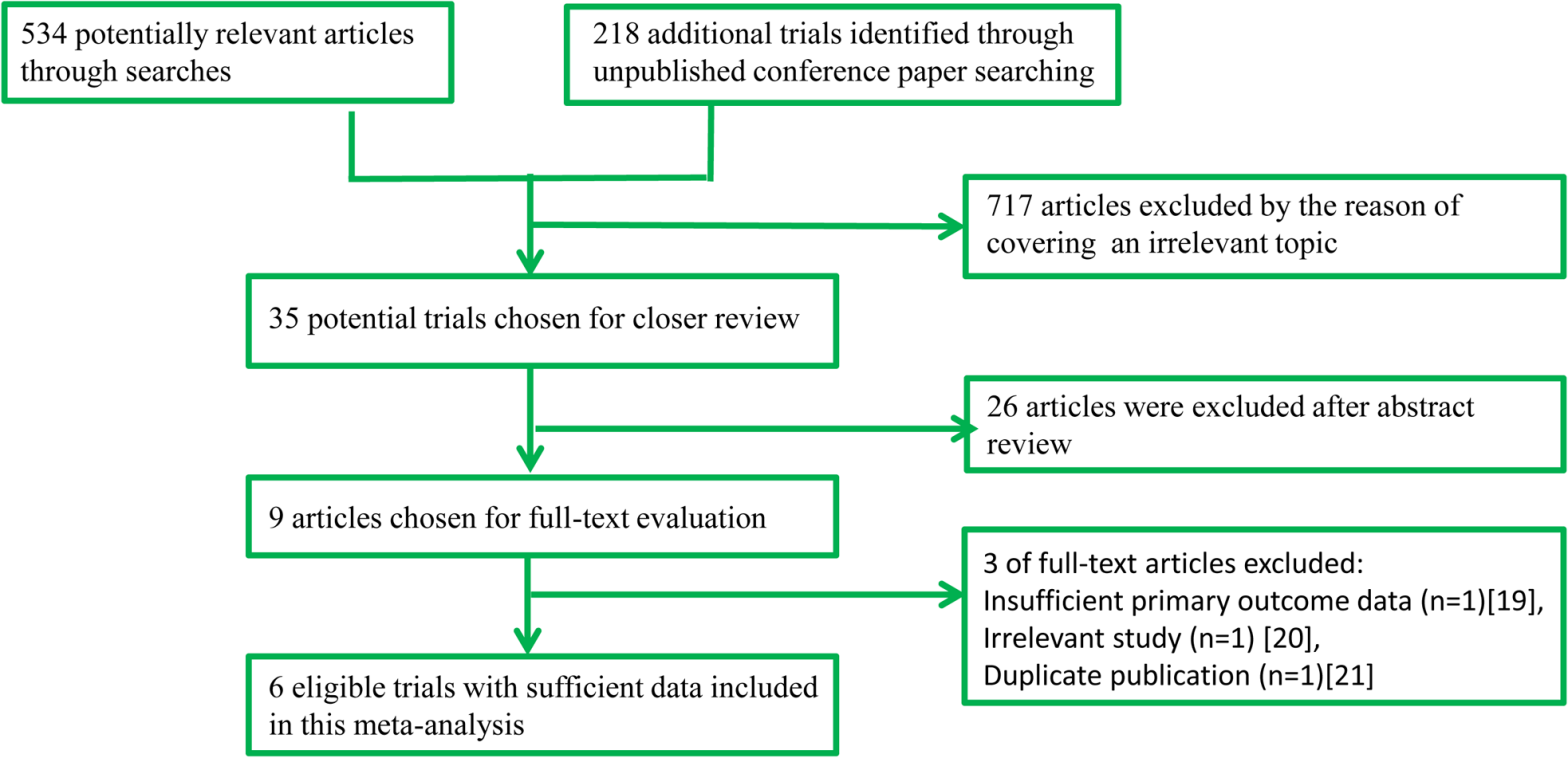
Flow diagram of study identification process.

A total of 752 potentially eligible studies were screened in the preliminary search. After screening the titles and abstracts, a total of 743 articles were excluded and 9 full manuscripts were investigated in detail. Based on the inclusion and exclusion criteria, 3 of those studies were excluded [19–21]. Therefore, 6 studies [12–15, 22, 23] (7080 participants) with more detailed and sufficient evaluations met our entry criteria and were retrieved for further analysis. A flow diagram of the study selection procedure is depicted in Fig 1. All included studies were published, peer-reviewed papers.

All 6 eligible articles evaluated the correlation between PBT and RC outcomes. The major characteristics of the 6 studies are summarized in Table 1. These studies were published from 2012–2014. The total number of enrolled patients was 7080, with individual samples ranging from 350–2895 (median 708). The reported mean age of the patients ranged from 67–69.5 years across the eligible studies. The follow-up period ranged from 18.7–132 months. The PBT ratio in each article ranged from 32.8% to 67%. Table 2 shows that 3 studies utilized disease recurrence, cancer-specific mortality and all-cause mortality to assess the prognostic value of PBT in patients [14, 21, 22]. Additionally, 1 study used cancer-specific survival and all-cause mortality [23], 1 study used progression-free survival and all-cause mortality [13], and 1 study used only all-cause mortality [15]. HRs and 95% CIs were directly obtained from these 6 studies. Quality assessment showed that the NOS score of each study was not less than 6, indicating that the methodological quality was generally good.

**Table 2:** Estimation of the hazard ratio.

Study	Survival analysis	Outcomes measured HR	Co-factors
**Sadeghi**	Cancer-specific mortality (multivariate)	not significant	age, chemotherapy use, pathologic stage, nodal status
	All-cause motility (multivariate)	not significant	
**Morgan**	All-cause motility (multivariate)	not significant	age, sex, race, preoperative hematocrit, comorbidity, pathologic stage, node density, margin status, estimated blood loss
**Linder**	Disease recurrence (multivariate)	not significant	age, sex, body mass index, preoperative hemoglobin level, estimate blood loss
	Cancer-specific mortality (multivariate)	significant	
	All-cause mortality (multivariate)	significant	
**Kluth**	Disease recurrence (multivariate)	not significant	age, gender, pathological grade, pathologic T and N stages, positive STSM, LVI, concomitant CIS, adjuvant chemotherapy
	Cancer-specific mortality (multivariate)	not significant	
	All-cause mortality (multivariate)	not significant	
**Gierth**	Progression-free survival (multivariate)	not significant	ASA score, age, tumor stage, information about preoperatively existing anemia, estimate blood loss
	All-cause mortality (multivariate)	significant	
**Abel**	Disease recurrence (multivariate)	not significant	age, sex, pathologic T and N stages, body mass index, preoperative hemoglobin level, diabetes, smoking
	Cancer-specific mortality (multivariate)	not significant	
	All-cause mortality (multivariate)	not significant	

Abbreviations: STSM, Soft tissue surgical margin; LVI, Lymphovascular invasion; CIS, Carcinoma in situ; ASA, American Society of Anesthesiologists

### 3.2 Primary outcomes

#### 3.2.1 Meta-analysis of all-cause mortality, cancer-specific mortality and recurrence

**Fig 2:**
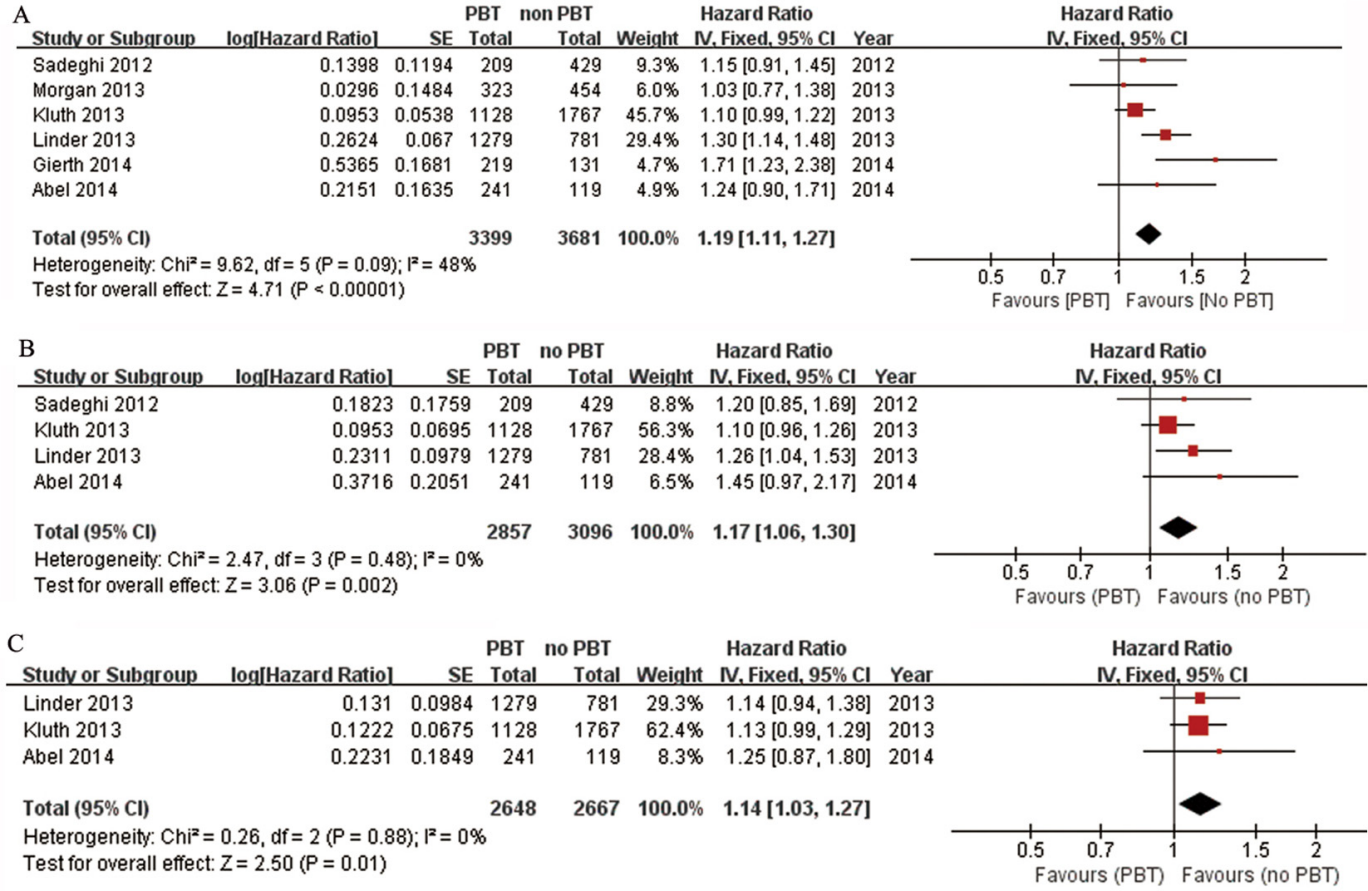
Fixed-effect model forest plots of HRs. (A) All-cause mortality, (B) Cancer-specific mortality, (C) Cancer recurrence. SE: standard error; CI: confidence interval; IV: inverse variance.

6 studies including 7080 patients were eligible for the final analysis [12–15, 22, 23]. A combined analysis of the relationship between PBT and all-cause mortality of patients undergoing RC is shown in forest plots (Fig 2). Our analysis suggested that PBT was associated with all-cause mortality after RC (HR: 1.19; 95% CI: 1.11–1.27; p<0.00001), with moderate heterogeneity between studies (I^2^ = 48%). Four studies (n = 5953) provided data on the association of PBT and cancer-specific mortality. We pooled the trial results in a meta-analysis and found a significant association between PBT and cancer-specific mortality, with an HR of 1.17 (95% CI: 1.06–1.30, P = 0.002; I^2^ = 0%, P = 0.48). Three studies (n = 5315) that evaluated cancer recurrence presented a significant difference in patients who received PBT (HR: 1.14; 95% CI: 1.03–1.27; I^2^ = 0%, P = 0.8) (Fig 2).

#### 3.2.2 Subgroup analysis of all-cause mortality

Subgroup analysis by the period of follow-up suggested that studies with a follow-up >30.8 months and ≤30.8 months had a significant impact on overall survival (>30.8: HR: 1.20, 95% CI: 1.11–1.30, I^2^ = 76%, P = 0.01; ≤30.8: HR: 1.13, 95% CI: 0.97–1.33, I^2^ = 0%, P = 0.73). When grouped according to the number of patients, studies with participants >708 and ≤708 suggested significant results (>708: HR: 1.16, 95% CI: 1.07–1.26, I^2^ = 56%, P = 0.0002; ≤708: HR: 1.29, 95% CI: 1.10–1.52, I^2^ = 47%, P = 0.15). We then focused on the median BT ratio in each study. The study with a >51.8% showed a significant impact on overall survival (HR: 1.34, 95% CI: 1.19–1.50, I^2^ = 21%, P = 0.28), but the study with a ≤51.8% BT ratio showed a slight impact on all-cause mortality (HR: 1.10, 95% CI = 1.00–1.21, I^2^ = 0%, P = 0.85) (Table 3).

**Table 3:** Subgroup analyses for all-cause mortality.

	No. of studies	No. of cases	Pooled HR (95% CI)	P value	Within-stratum heterogeneity
**Follow-up (months)** *					
**>30.8**	3	5305	1.20 (1.11–1.30)	<0.00001	I2 = 76%, p = 0.01
**≤30.8**	3	1775	1.13 (0.97–1.33)	0.12	I2 = 0%, p = 0.73
**No. of patients** **					
**>708**	3	5732	1.16 (1.07–1.26)	0.0002	I2 = 56%, p = 0.11
**≤708**	3	1348	1.29 (1.10–1.52)	0.002	I2 = 47%, p = 0.15
**PBT ratio** ***					
**>51.8%**	3	2770	1.34 (1.19–1.50)	<0.00001	I2 = 21%, p = 0.28
**≤51.8%**	3	4310	1.10 (1.00–1.21)	0.04	I2 = 0%, p = 0.85

HR: Hazard ratio; CI: Confidence interval

* Median follow-up period of included studies: 30.8 months

**Median patients’ number of included studies: 708

***Median PBT ratio of included studies: 51.8%

### 3.3 Sensitivity analysis and publication bias

A sensitivity analysis was conducted, in which one study was deleted at a time, to gauge the stability of our results. The corresponding pooled HR for all-cause mortality did not significantly change (HR was between 1.17 and 1.26), which suggests that the result was robust.

**Fig 3:**
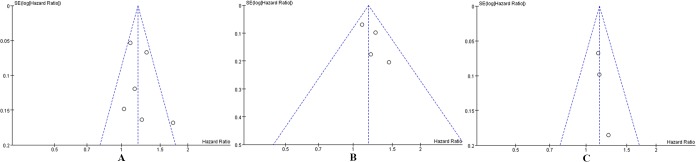
Funnel plots for publication bias in studies of PBT in patients with bladder cancer who underwent RC. (A) All-cause mortality. (B) Cancer-specific mortality. (C) Cancer recurrence.

Publication bias was tested in this meta-analysis using funnel plots. Fig 3 shows that the funnel plots presented no proof of obvious publication bias in any of the included studies for all-cause mortality or cancer-specific mortality or recurrence, which suggests no publication bias.

## Discussion

To the best of our knowledge, this is the first meta-analysis that has been conducted on the impact of PBT on mortality and recurrence in patients with bladder cancer who underwent RC. The present meta-analysis combined the outcomes available in 6 published studies and concluded that PBT was associated with significantly increased risks of all-cause mortality (HR: 1.19, 95% CI: 1.11–1.27, P<0.00001) and worse cancer-specific mortality (HR: 1.17, 95% CI: 1.06–1.30, P = 0.002) and recurrence (HR: 1.14, 95% CI: 1.03–1.27, P = 0.01). Additionally, the sensitivity analysis showed that the significant association between PBT and increased mortality and cancer recurrence remained regardless of whether one of the included studies was omitted, which suggests the robustness of this result. A quality assessment was performed independently and reproducibly by two authors according to Newcastle-Ottawa guidelines. We ensured that the included studies were high quality by comprehensively and scientifically evaluating the articles.

Numerous reasons may explain why PBT was associated with an increased risk of death and recurrence in patients who underwent surgical resection of cancer. One hypothesis underlying this association is the possible immunosuppressive effect of PBT [24, 25]. The infusion of foreign antigens in transfused blood products induces immune suppression, anergy and clonal deletion in studies in experimental animals [26], which may predispose already immunosuppressed cancer patients to tumor cell spread and facilitate tumor growth and reduced survival. However, most studies that have evaluated proposed mechanisms have been performed in rodents, and these findings may not be applicable to the human immune system [27]. Other possible mechanisms that have been postulated to explain the noted association between PBT and survival include the increased incidence of postoperative infection and blood type incompatibility [28].

The baseline characteristics of patients might have affected the conclusions of each included report. Therefore, further subgroup analyses were stratified using patient number, follow-up duration and PBT ratio. A significant association was observed between PBT and increased mortality when the follow-up period was more than 39 months. This finding should be interpreted cautiously as a worse long-term survival of PBT, which was shown in several studies [29–31], but not in RC of bladder cancer. Another explanation may be due to increased mortality with prolonged follow-up times. PBT ratios ranged from 32.8%-67% in the 6 included studies. The subgroup analysis by BT ratio revealed that the increased all-cause mortality occurred when the BT ratio was high. These differences in transfusion ratios may be explained by several reasons, including different patient populations and differences in traditions and norms. If the significant institutional-level differences of the patients are ignored, we believe that the higher rate of BT (62.8% in the ≥51.8% subgroup and 43.3% in the <51.8% subgroup) may cause by unnecessary blood transfusion, which may increase the all-cause mortality of patients who underwent RC (HR: 1.34, 1.19–1.50, P<0.05, vs. HR: 1.10, 1.00–1.21, P<0.05, respectively). Perioperative blood loss is an important operative complication in patients undergoing major surgery, which may increase the chance of PBT, subsequent postoperative morbidity and mortality [32]. Therefore, individual centers should pay attention to their current transfusion practice and reduce intraoperative blood loss as much as possible.

The timing of blood transfusion is also an important factor for clinical outcomes. Gierth et al. showed that intraoperative and postoperative blood transfusions independently influence progression-free survival and overall survival [13]. Abel et al. combined outcomes from two independent cohorts of consecutive patients with bladder cancer treated with RC and demonstrated that intraoperative BT (HR: 1.41, 95% CI: 1.22–1.63, P<0.00001), but not postoperative BT (HR: 1.04, 95% CI: 0.87–1.24, P = 0.65), was associated with an increased risk of bladder cancer all-cause mortality [22].

The present meta-analysis has limitations. First, the most important limitation is that all of the included studies were retrospective samples rather than randomized controlled trials, and the clinicians could not be blinded to the selection of patients who received BT. Multivariable models were used in the included studies, but the adjusted cofounders were not the same for the adjusted HRs (Table 3). Therefore, we were unable to conduct stratified analyses based on possible confounders, such as preoperative hemoglobin levels or perioperative blood losses. Some studies demonstrated that estimated blood loss was an independent factor to predict survival or recurrence after surgery [33, 34], but only 3 of the 6 studies in this meta-analysis considered intraoperative blood loss [13, 15, 21]. Several reports suggested that intraoperative blood loss during surgery for cancer is a critical risk factor of mortality and recurrence. Notably, Linder et al. repeated their multivariate analysis to include the variable of estimate blood loss in response to this issue, and they discovered that PBT remained significantly associated with increased risks of cancer-specific mortality (HR: 1.26; P = 0.017) and all-cause mortality (HR: 1.30; P = 0.0002) [21]. Second, studies performed with positive results or significant outcomes are more apt to be published, which suggests a potential publication bias. The sample sizes of enrolled research (from 350 to 2,895) varied widely; therefore, the statistical power or weight of each study was greatly different, which inevitably causes bias to varying degrees. In addition, one report did not provide sufficient data and was excluded from our analysis [19]. Third, reports in languages other than English were excluded, so a potential language bias may be present in our meta-analysis.

The results of our meta-analysis confirmed that PBT might be an independent predicative factor of mortality and disease recurrence in bladder cancer patients who undergo RC. Autotransfusion is safer than ABT [35], but the amount of phlebotomized blood is insufficient to completely replace blood loss. Substantial variation exists in the hospital-level use of PBT for patients undergoing RC, which might be related to different rates of cancer recurrence and all-cause mortality for these patients. Clinicians should reconsider the use of blood conservation methods to avoid transfusions, especially in patents who require limited amounts of transfused blood products. Further studies are needed to investigate a standard to determine whether a patient requires a BT. Regardless, every effort should be made to reduce intraoperative blood loss that requires BT because it may be associated with a decrease in long-term survival. The conclusions of this study should be interpreted with caution based on the study limitations. Future large studies with rigorously designed methodologies are warranted to confirm our results.

## Supporting Information

S1 PRISMA Checklist PRISMA 2009 Checklist. (DOC)
